# Effect of *Salvadora persica* on resin-dentin bond stability

**DOI:** 10.1186/s12903-024-04244-3

**Published:** 2024-04-29

**Authors:** Manar M. Abu-Nawareg, Hanan K. Abouelseoud, Ahmed Z. Zidan

**Affiliations:** 1 Department of Restorative Dentistry, Faculty of Dentistry, King Abdulaziz University, P.O. Box 80209, Jeddah, 21589 Saudi Arabia; 2 Biomaterials Department, Faculty of Dentistry, Cairo University, Cairo, Egypt; 3 Operative Dentistry Department, Faculty of Dentistry, Cairo University, Cairo, Egypt; 4 Restorative Dentistry Department, Faculty of Dentistry, Umm Al-Qura University, Mekkah, Saudi Arabia; 5 Biomaterials Department, Faculty of Dentistry, October University for Modern Sciences and Arts (MSA), Cairo, Egypt

**Keywords:** Natural crosslinker, *Salvadora Persica*, Microtensile bond strength, Dentin stiffness, Enzymatic degradation

## Abstract

**Background:**

The stability of resin–dentin interfaces is still highly questionable. The aim of this study was to evaluate the effect of *Salvadora persica* on resin–dentin bond durability.

**Materials and methods:**

Extracted human third molars were used to provide mid-coronal dentin, which was treated with 20% *Salvadora persica* extract for 1 min after acid-etching. Microtensile bond strength and interfacial nanoleakage were evaluated after 24 h and 6 months. A three-point flexure test was used to measure the stiffness of completely demineralized dentin sticks before and after treatment with *Salvadora persica* extract. The hydroxyproline release test was also used to measure collagen degradation by endogenous dentin proteases. Statistical analysis was performed using two-way ANOVA followed by post hoc Bonferroni test and unpaired *t*-test. *P*-values ***<*** 0.05 were considered statistically significant.

**Results:**

The use of *Salvadora persica* as an additional primer with etch-and-rinse adhesive did not affect the immediate bond strengths and nanoleakage (*p* > 0.05). After 6 months, the bond strength of the control group decreased (*p* = 0.007), and nanoleakage increased (*p* = 0.006), while *Salvadora persica* group showed no significant difference in bond strength and nanoleakage compared to their 24 h groups (*p* > 0.05). *Salvadora persica* increased dentin stiffness and decreased collagen degradation (*p* < 0.001) compared to their controls.

**Conclusion:**

*Salvadora persica* extract pretreatment of acid-etched dentin preserved resin–dentin bonded interface for 6 months.

**Clinical significance:**

Durability of resin-dentin bonded interfaces is still highly questionable. Endogenous dentinal matrix metalloproteinases play an important role in degradation of dentinal collagen within such interfaces. Salvadora persica may preserve resin-dentin interfaces for longer periods of time contributing to greater clinical success and longevity of resin composite restorations.

## Introduction

The durability of adhesive resin composite restorations is highly dependent on the stability of resin–dentin interfaces [1]. Adhesion to dentin depends mainly on hybrid layer formation, which is a resin-infiltrated demineralized dentinal collagen layer [2]. The quality of such a layer is a major factor that controls the longevity of the restoration [3].

Degradation of unprotected collagen within the hybrid layer by dentinal matrix metalloproteinases (MMPs) is highly responsible for the destruction of the resin–dentin bonded interface [3–5]. MMPs are bonded to collagen fibrils and trapped by apatite minerals. The acidity of both etch-and-rinse and self-etch adhesives dissolves apatite crystallites, exposing and activating dentin MMPs, which are responsible for collagen degradation [3–5].

Previous studies provided different approaches aiming to inhibit collagen degradation by MMPs to improve the stability of resin–dentin interfaces [2, 6–11]. Crosslinking agents were used to increase the stiffness of collagen and increase its degradation resistance through the formation of additional hydrogen bonding and/or intra- and intermolecular covalent bonds [2, 10–16]. Several crosslinking agents were used in previous studies [10–22], but the optimum MMP inhibitory effect has not yet been reached.

Nowadays, there is an ongoing interest in naturally occurring crosslinkers that may improve resin–dentin bonded interface durability [14–16]. Phytotherapy is one of the recently invaded fields. It is considered a return to nature where plant products and extracts are tested for therapeutic purposes [23]. Epigallocatechin-3-gallate, extracted from green tea, was efficient in enhancing resin-dentin bond durability by crosslinking dentinal collagen and thus inactivating MMPs [24–26]. Miswak chewing sticks are natural toothbrushes obtained from the *Salvadora persica* plant (Arak tree). The use of miswak predates the inception of Islam. It was a pre-Islamic custom used by the ancient Arabs to whiten their teeth and make them shiny [27–29]. The World Health Organization (WHO) recommended the use of miswak in 1986 as an efficient oral hygiene tool [30]. Miswak or *Salvadora persica* extract has several biological properties, such as antibacterial, antifungal, anti-inflammatory, antioxidant, analgesic, and anticariogenic effects [23, 28]. Moreover, *Salvadora persica* extract was reported to include tannins and vitamin C [29, 31, 32], where both have a crosslinking effect on collagen fibrils [33, 34]. However, the effect of *Salvadora persica* extract in preserving dentinal collagen to improve resin–dentin bonded interface stability has not been fully investigated. Therefore, can *Salvadora persica* preserve resin-dentin interfaces for longer periods of time?

The objective of the current study was to evaluate the effect of 20% *Salvadora persica* extract on microtensile bond strength and nanoleakage of resin–dentin interfaces created by two-step etch-and-rinse adhesive after 24 h and 6 m of water storage. Stiffness and hydroxyproline release tests were also performed to evaluate the potential reinforcing and preserving effect of *Salvadora persica* extract on dentinal collagen. The tested null hypotheses were that the use of *Salvadora persica* extract (1) has no effect on immediate bond strength and nanoleakage, (2) has no preserving effect on the bonded interface over time, (3) has no reinforcing effect on dentin, and (4) has no preserving effect on dentinal collagen by MMPs inactivation.

## Materials and methods

### Preparation of 20% *Salvadora persica* aqueous extract

A fresh 1 kg of *Salvadora persica* roots (Miswak sticks) was selected by an expert on the plant and was obtained from a store in Jeddah, Saudi Arabia. The roots were originally collected from Almakhwah, 45 km west of Albahah, south of Saudi Arabia (1947′0″ N, 4126′0″ E). Extract of *Salvadora persica* was prepared according to a protocol published in previous studies [28, 35, 36]. The roots were cleaned, washed with distilled water, dried for a few days at room temperature, then cut into tiny pieces and ground in a ball mill to produce a powder. A 20 gm of *Salvadora persica* powder was added to 100 mL of deionized sterile water and was left at 4 °C for 48 h. The mixture was then centrifuged for 10 min at 2200× *g* rpm, producing a 20% *Salvadora persica* aqueous extract. Ultraviolet light (Camag UV-Cabinet II, Basel, Switzerland) delivering ∼ 10 mW/cm^2^ at 366 nm was used for 1 h to sterilize the extract, then it was stored at 4 °C and was used within 1 week from its preparation [28].

### Microtensile bond strength test and nanoleakage analysis

A total of 20 extracted sound human third molars were obtained with the informed consent of the donor following a protocol (# 004–15) approved by the Research Ethics Committee, King Abdulaziz University, Jeddah, Saudi Arabia. The teeth were stored for 1 week in 0.5% chloramine T solution (Sigma-Aldrich Co., St. Louis, MO, USA), then in distilled water at 4 °C till use within 2 months following extraction. A low-speed diamond saw (Micromet AG, Munich, Germany) under water coolant was used to cut occlusal enamel and superficial dentin in a direction perpendicular to the long axis of the tooth. The exposed middle dentinal surfaces were polished with wet 320-grit silicon carbide paper (Norton Saint-Gobain Abrasives, Worcester, MA, USA) for 60 s to remove any enamel remnants and create standardized smear layers.

### Restorative procedure

The smear layer-covered dentinal surfaces of the 20 teeth used were etched for 15 s using Scotchbond Universal etching gel (3 M ESPE, St. Paul, MN, USA, Lot #: 7,936,326, composition: 30–40 wt% % phosphoric acid, water, silica, polyethylene glycol, aluminium oxide), rinsed with water for 10 s, then blot-dried using cotton pellets and kept moist without pooling following the wet bonding technique. The 20 teeth, with etched dentinal surfaces, were divided into 2 groups (*n* = 10) according to the dentin pretreatment, which was as follows: Group 1: Control, etched dentinal surfaces were bonded by Adper Single Bond 2 (3 M ESPE, St. Paul, MN, USA, Lot #: NC68418, composition: BISGMA, HEMA, dimethacrylate, methacrylate-modified polyalkenoic acid copolymer, 10 wt% 5 nm silica particles, ethyl alcohol, water, initiators, other name: Adper Scotchbond 1 XT) according to the manufacturer’s instructions, without any dentin pretreatment as follows: two consecutive coats of the adhesive were applied to etched dentin for 15 s with gentle agitation using a fully saturated microsponge (Pearson Dental Supply, CA, USA). Gentle adhesive air-thinning for 5 s was performed then the adhesive was light-cured for 10 s (Light Emitting Diode curing unit, 3 M ESPE Elipar, Seefeld, Germany delivering 1200 mW/cm^2^ at 430–480 nm); Group 2: *Salvadora persica* extract was applied generously using a fully saturated microsponge (Pearson Dental Supply, CA, USA) to the etched dentinal surface with gentle agitation for 1 min, water rinsed for 15 s, blot-dried, then was finally bonded with Adper Single Bond 2 adhesive as mentioned in group 1.

Resin-based composite (Filtek Z350 XT, 3 M ESPE, St. Paul, MN, USA, Lot #: NF30942) build-up is performed using the incremental technique where each increment was light-cured (Light Emitting Diode curing unit, 3 M ESPE Elipar, Seefeld, Germany delivering 1200 mW/cm^2^ at 430–480 nm) for 20 s. All samples were placed in distilled water for 24 h at 37 °C. Each tooth was then cut parallel to its long axis through the resin–dentin interface, producing 16 sticks (1 × 1 mm ± 0.1 mm) per tooth [2, 9, 10, 37], using a Techcut 4 diamond saw (Allied High Tech Products Inc., Compton, CA, USA). The sticks produced from each tooth were stored separately in distilled water at 37 °C. Each group (*n* = 10) was divided into 2 subgroups according to the storage interval (*n* = 5): 24 h and 6 m.

### Microtensile bond strength test

After storage either for 24 h or 6 m, 15 sticks from each of the 5 teeth of each subgroup were used for the microtensile bond strength test. A digital caliper (Dasqua tools, Chengdu, China) was used to record the dimensions of each stick accurately to 0.01 mm. A microtensile tester (Bisco Inc., Schaumburg, IL, USA) was used to apply a tensile force at the bonded interface of each stick at a crosshead speed of 1 mm/min until failure. Microtensile bond strength (MPa) was calculated for each stick. The average of the bond strength values of the 15 sticks of each tooth was calculated to provide a single value for each tooth. Then, the values of the 5 teeth of each subgroup were averaged to provide a grand mean for each subgroup (the statistical unit is the tooth) [9, 10]. Fracture mode of all debonded specimens was examined at 50x magnification using a stereomicroscope (Meiji Techno Co., Ltd., Tokyo, Japan) and was classified into adhesive (A), cohesive in dentin (CD), cohesive in composite (CC) or mixed (M) failures.

### Nanoleakage evaluation

**Fig. 1:**
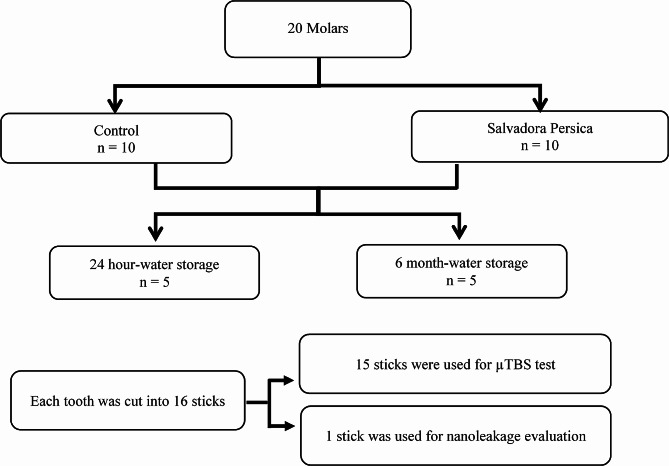
Experimental design for microtensile bond strength test and nanoleakage analysis. For bond strength test, there were 2 groups (control and *Salvadora persica*). Each group contained 10 teeth, which were divided into 2 subgroups (24 h and 6 m). Each subgroup contained 5 teeth or 75 (5 × 15) sticks. The values of the 15 sticks per tooth were averaged to provide a single value for each tooth. Then, a grand mean was obtained from the values of the 5 teeth of each subgroup (the tooth is the statistical unit). For nanoleakage test, one stick from each tooth of each subgroup was used for nanoleakage analysis (*n* = 5). Two images were selected from each stick, where the amount of silver nitrate was calculated in each image then averaged to give a single nanoleakage value for each stick. The nanoleakage values of the 5 sticks per subgroup were averaged to provide a mean nanoleakage % value for each time period in each group

One stick from each tooth of each subgroup was used for nanoleakage analysis (*n* = 5). The sticks were prepared according to the technique described by Tay et al. [38]. The sticks were completely coated by 2 layers of nail varnish (Shenzhen Meixin Industry Co., Ltd., Guandong, China), except for 1 mm at the interface, which was left uncoated. A solution of ammoniacal silver nitrate (50 wt%, pH 9.5) was used to soak the sticks for 24 h, followed by rinsing, and then placed for 8 h in a photodeveloper (Kodak Professional T-Max developer, Kodak Alaris Inc., Rochester, NY 14,615, USA) under fluorescent light. The sticks were polished with wet silicon carbide papers (Norton Saint-Gobain Abrasives, Worcester, MA, USA) of increasing fineness (600–1200-grit), followed by soft polishing cloth with 0.05 mm alumina particles suspension (Buehler, Lake Bluff, IL, USA) and then ultrasonically cleaned (Ultrasonic Cleaning System 2014, L&R Manufacturing, Kearny, NJ, USA) for 5 min. Scanning electron microscopy of resin–dentin interfaces was performed (SEM; Quanta 200 ESEM, FEI France, Mérignac, France) using backscattered mode at 1000x. The amount of silver nitrate precipitated within the interface was quantitated using image analysis software (Version 1.32 NIH Image, Scion Corp., Fredrick, MD, USA) [10, 38]. Figure 1 shows the experimental design for microtensile bond strength test and nanoleakage analysis.

### Stiffness and hydroxyproline release tests

A total of 40 third molar teeth were selected as discussed before and were sectioned to provide 0.5 mm thick discs of mid coronal dentin, which were further cut into 40 dentin sticks (width = 3 mm, length = 6 mm). The sticks were placed in 10% phosphoric acid in vibrating sealed bottles for 18 h at 4 °C until they were completely demineralized and then rinsed with distilled water for 2 h. The modulus of elasticity of each stick was measured and was set at 5 MPa to ensure complete dem ineralization [39]. The 40 completely demineralized dentin sticks were equally and randomly divided into 2 groups (20 each) according to the test evaluated: stiffness and hydroxyproline tests.

### Stiffness evaluation

A three-point flexure test was used to measure the initial stiffness of 20 completely demineralized dentin sticks. An aluminum testing jig with a 2.5 mm support span was used and specimens were centrally subjected to compression while immersed in distilled water, using a 1000 g load cell testing machine (Vitrodyne 1000, Burlington, VA, USA) at 1 mm/min crosshead speed. The specimens were deformed to a maximum strain of 15%. Load-displacement curves were produced and were then converted to stress-strain curves. Elastic modulus (E) was calculated at the steepest, most linear portion of stress–strain curves using the following formula:

$${\rm{E}}\,{\rm{ = }}\,{\rm{m}}{{\rm{L}}^{\rm{3}}}{\rm{/4b}}{{\rm{d}}^{\rm{3}}}$$

where m = slope (N/mm); L = support span (mm); d = thickness of stick (mm); b = width of stick (mm).

Since specimen displacement was not measured with an extensometer or strain gauge but was an estimate from cross-head displacement, and the specimens’ thickness was not one-sixteenth of the length [40], the calculated elastic moduli were approximate. We were more interested in changes in modulus of elasticity rather than their absolute values.

After measuring the initial stiffness, the 20 demineralized sticks were immersed in distilled water (control) or *Salvadora persica* extract for 1 min (*n* = 10) and then immediately subjected again to 3-point flexure testing, under same parameters, to calculate the new stiffness values [13].

### Hydroxyproline release test

Hydroxyproline is a major constituent of dentinal collagen. Degradation of collagen can be detected by the amount of hydroxyproline released [41]. When demineralized dentin is placed in a buffer solution at 37 °C, dentinal MMPs degrade the collagen network, which reduces its stiffness and results in loss of dry mass containing hydroxyproline [13, 42]. However, crosslinking the demineralized dentin resulted in a significant reduction in the lost dry mass, thus reducing the amount of hydroxyproline release [11, 42].

**Fig. 2:**
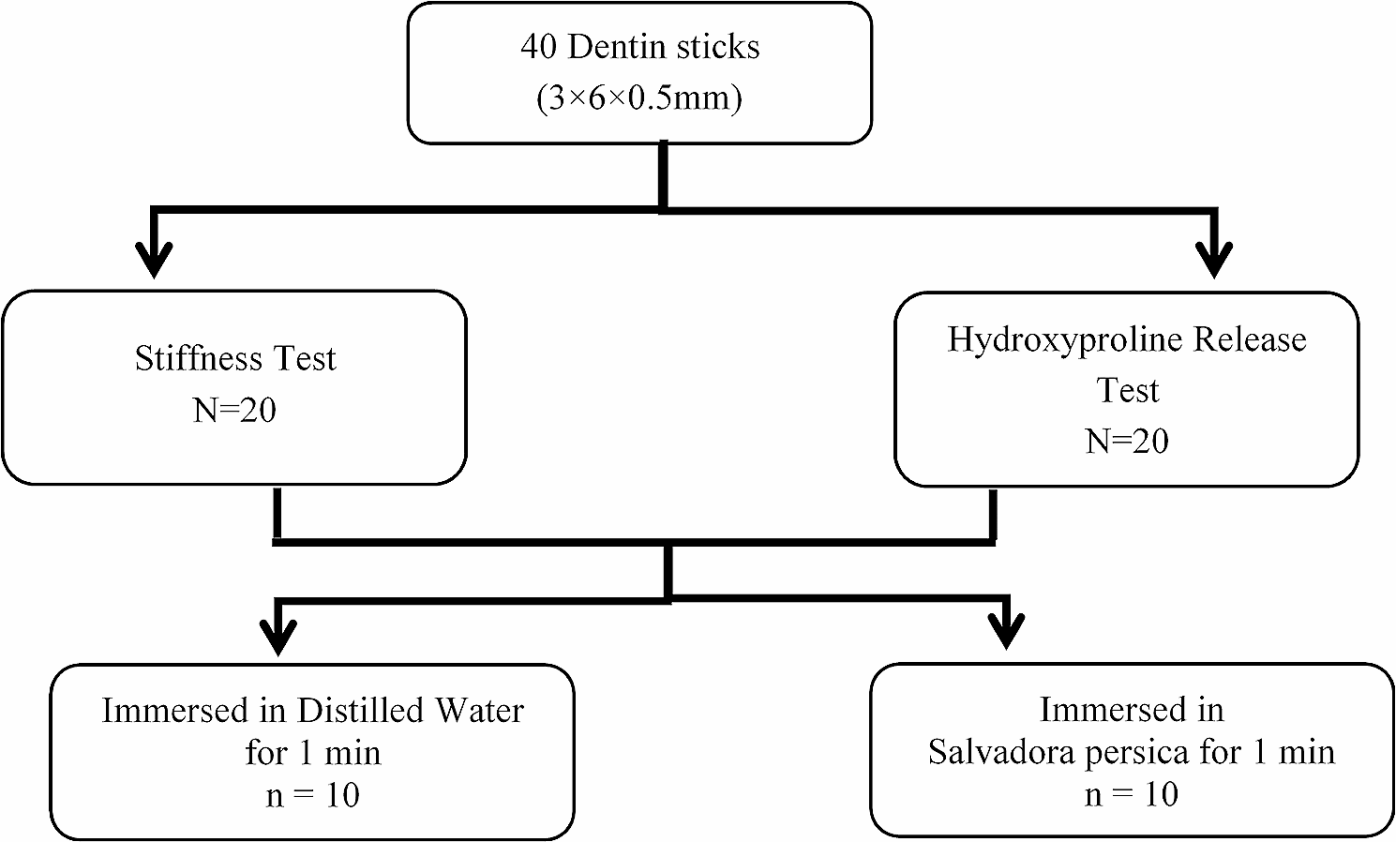
Experimental design for stiffness and hydroxyproline release tests, (*n* = 10)

For this test, 20 dentin sticks (width = 3 mm, length 6 mm, thickness = 0.5 mm) were prepared and completely demineralized, as mentioned before. The demineralized sticks were placed in distilled water or *Salvadora persica* extract for 1 min (*n* = 10). Hydroxyproline release was measured using a colorimetric method [13, 43]. In brief, the treated sticks were immersed in a buffer solution (0.05 M HEPES solution, pH 7.4, Mallinckrodt Baker, Inc. Phillipsburg, NJ, USA) at 37 °C for 1 week. Hydrochloric acid (6 N HCL, Arcos Organics, Phillipsburg, NJ, USA) was used to hydrolyze the dissolved collagen into amino acids. Then, the HCL was allowed to evaporate, leaving dry remnants, which were then examined for the amount of hydroxyproline released. Figure 2 shows the experimental design for stiffness and hydroxyproline release tests.

### Statistical analysis

Data were collected and entered using the statistical package for the Social Sciences (SPSS) version 28 (IBM Corp., Armonk, NY, USA). Normality was checked for by the Shapiro–Wilk test and proved to not deviate from normal distribution (*p*-value > 0.05). Data were summarized using means and standard deviations. For the microtensile bond strength test and nanoleakage assessment, two-way ANOVA was used to compare treatment and time interval groups, followed by the post hoc Bonferroni test. For stiffness and hydroxyproline release tests, a comparison between groups was performed using an unpaired *t*-test. *p*-values < 0.05 were considered as statistically significant.

## Results

### Microtensile bond strength

Table 1 shows the microtensile bond strength values exhibited by the *Salvadora persica* group versus the control group at 24 h and 6 m storage periods.

**Table 1:** Mean values ± standard deviations (SDs) of microtensile bond strength (µTBS) in MPa for both experimental groups at the 2 storage intervals

Storage Time	Control	Salvadora persica	*p*-Value
24 h	43.2 ± 6.6 ^Aa^ 34.6 A/2.6CD,1.3CC,61.3 M	42.9 ± 7.03 ^Aa^ 30.6 A,4CD,5.3CC,60 M	0.941
6 m	30.7 ± 5.15 ^Bb^ 52 A,1.3CD,4CC,42.6 M	41.3 ± 6.43 ^Aa^ 37.3 A,2.6CD,4CC,56 M	0.018 *
*p*-value	0.007 *	0.695	

Different uppercase letters indicates a significant difference (*p* < 0.05) in the same column, while different lowercase letters indicates a significant difference (*p* < 0.05) in the same row. * statistically significant at *p* < 0.05, (*n* = 5). Fracture mode percentages were classified into: A, Adhesive; CD, cohesive in dentin; CC, cohesive in composite; M, Mixed failure

At 24 h, microtensile bond strength values obtained by *Salvadora persica* did not differ significantly from the control group (*p =* 0.941). After 6 m of water storage, the control group showed a significant drop in bond strength when compared to the 24 h group (*p =* 0.007). On the other hand, there was no significant difference in bond strength values between the two testing periods for the *Salvadora persica* group (*p =* 0.695). Moreover, at the 6 m storage period, the bond strength exhibited by the *Salvadora persica* group was significantly higher than that of the control group (*p* = 0.018).

### Nanoleakage

Nanoleakage% values of the *Salvadora persica* group versus the control group, at 24 h and 6 m, are shown in Table 2.

**Table 2:** Mean values ± standard deviations (SDs) of nanoleakage (%) of the 2 experimental groups at both storage intervals

Storage Time	Control	Salvadora persica	*p*-Value
24 h	54.6 ± 9.79	53.8 ± 7.37	0.869
6 m	69.7 ± 7.32	57.6 ± 4.84	0.022 *
*p*-value	0.006 *	0.437	

* statistically significant at *p* < 0.05, (*n* = 5)

**Fig. 3:**
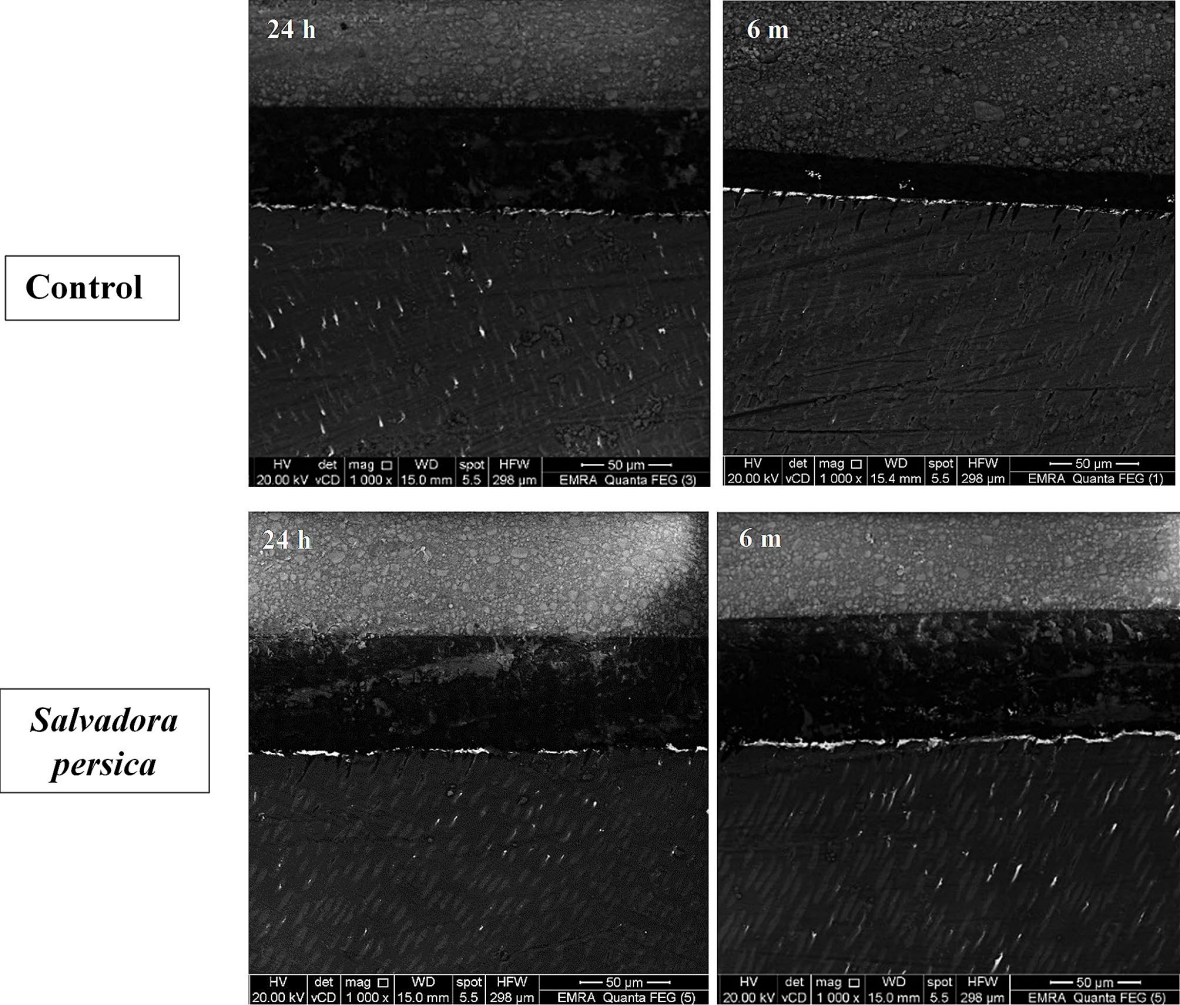
Representative scanning electron microscope (SEM) images of resin–dentin interfaces demonstrating interfacial nanoleakage for the control and *Salvadora persica* groups after 24 h and 6 m storage periods. Magnification: 1000x

After 24 h water storage, the two tested groups showed no significant difference in nanoleakage% (*p* = 0.869). After 6 m, the control group showed a significant increase in nanoleakage when compared to the 24 h group (*p* = 0.006). On the other hand, there was no significant difference in nanoleakage between the two testing periods for the *Salvadora persica* group (*p* = 0.437). Furthermore, at a 6 m storage period, the nanoleakage% of the control group was significantly higher than the *Salvadora persica* group (*p* = 0.022). Representative SEM pictures of nanoleakage % of different groups at 1000× magnification are shown in Fig. 3.

### Stiffness

**Fig. 4:**
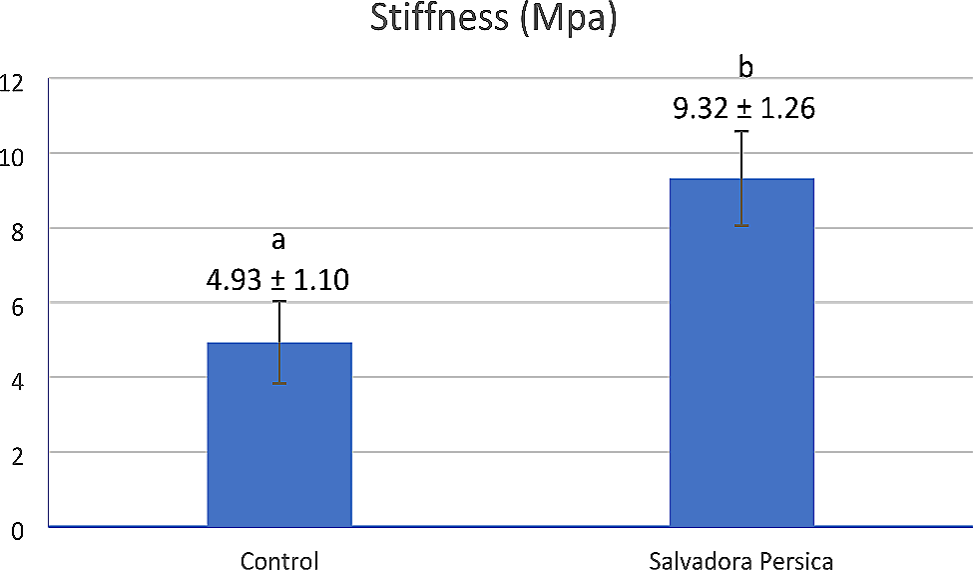
Bar chart presenting mean values and standard deviations for stiffness of completely demineralized dentin sticks subjected to 3-point flexure after immersion in water (control) or *Salvadora persica* extract for 1 min (*n* = 10). Different lowercase letters indicate significant differences at *p* < 0.05

The stiffness values (MPa) of completely demineralized dentin sticks of the control and the *Salvadora persica* groups are presented in Fig. 4. *Salvadora persica* group showed a significantly higher stiffness (9.32 ± 1.26 MPa) value than the demineralized beams of the control group, which were kept in water without any treatment (4.93 ± 1.10) (*p* < 0.001).

### Hydroxyproline release test

**Fig. 5:**
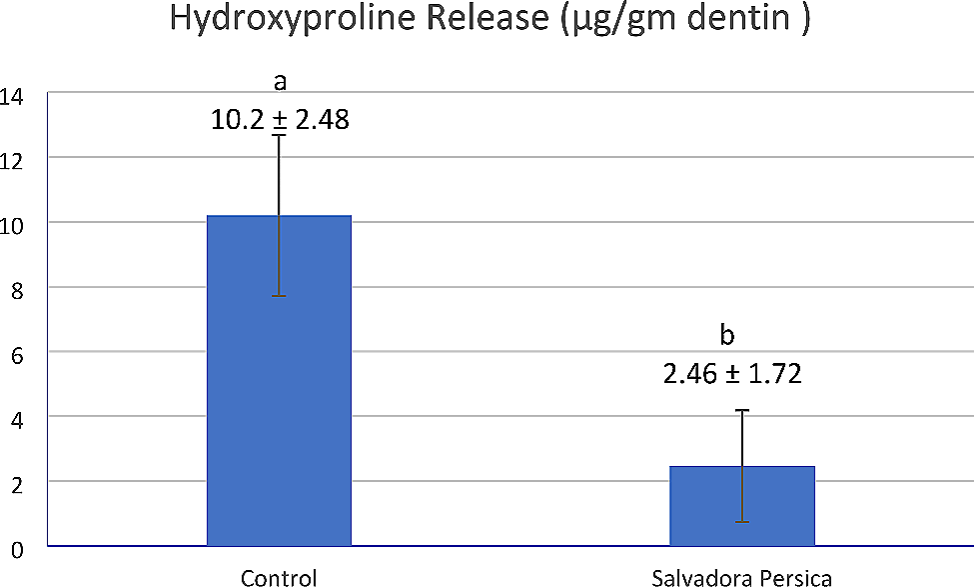
Bar chart presenting mean values and standard deviations for hydroxyproline release from demineralized dentin sticks immersed in water (control) or *Salvadora persica* extract for 1 min, rinsed, and then stored in buffer solution for 1 week at 37 °C with vibration (*n* = 10). Different lowercase letters indicate significant differences at *p* < 0.05

Figure 5 presents the amount of hydroxyproline released in µg/gm dentin for *Salvadora persica* and control groups. Hydroxyproline release of the control group was significantly higher (10.2 ± 2.48 µg/gm dentin) than the *Salvadora persica* group (2.46 ± 1.72 µg/gm dentin) (*p* < 0.001)

## Discussion

Several approaches were used to enhance resin–dentin bond stability either by inhibiting or inactivating matrix metalloproteinases [1, 8, 9]. Chlorhexidine (CHX) is proven to serve as a potent MMP inhibitor, preserving resin–dentin bonded interfaces. Unfortunately, their long-term effect was doubtful due to the possibility of their leaching out, leaving the bonded interface unprotected and prone to degradation [6, 9, 19]. Furthermore, several crosslinking agents were used previously to crosslink the dentinal collagen fibrils, increasing their degradation resistance to MMPs [10–16]. Crosslinking agents such as glutaraldehyde, proanthocyanidin (grape seed extract), riboflavin, and, more recently, chitosan were used [10, 13, 17, 19].

Although glutaraldehyde is commonly used to crosslink dentinal collagen, proanthocyanidin is considered a better option due to the lack of toxic effects that are reported with glutaraldehyde. However, proanthocyanidin has an undesirable staining effect on dentin. Furthermore, riboflavin is reported to be a potent collagen crosslinking agent due to its lack of toxicity; however, it is more efficient when activated by ultraviolet light, which is not common in all dental clinics [10, 12, 13]. Many studies used Epigallocatechin-3-gallate, present in green tea, as a naturally occurring crosslinker which showed promising results in reducing collagen degradation by MMPs and improving resin-dentin bonded interfaces stability [24–26]. On the other hand, the use of the naturally occurring *Salvadora persica* as a potential natural crosslinker may provide a great solution for enhancing resin–dentin bond durability; however, its effect on dentinal collagen is not yet fully investigated.

Besides the crosslinking effect of *Salvadora persica* extract and the possible bond durability enhancement, it has many advantageous qualities that may be beneficial to esthetic adhesive restorations. *Salvadora persica* has antibacterial, antifungal, anti-inflammatory, analgesic, and anticariogenic effects [23, 28, 32]. Therefore, if used, it may reduce or eliminate bacteria from cavity preparations, may have a sedative effect on the pulp, and may reduce the possibility of recurrent caries.

The results of the current study demonstrated a significant deterioration in microtensile bond strength (*p* = 0.007) and nanoleakage (*p* = 0.006) for the control group over a 6-month storage period. This was in agreement with many other studies [2, 5, 7, 9, 10, 34, 44]. The stability of resin–dentin bonded interfaces has been compromised due to hybrid layer degradation [1, 3] caused by many factors [1, 9], including improper resin penetration into the etched dentinal layer and hydrophilicity of adhesive resins [9, 45] as well as collagenolytic activity of endogenous MMPs and cysteine cathepsins [2–12].

The current study used *Salvadora persica* in an attempt to crosslink dentinal collagen at the bonded interface to increase its resistance to degradation by endogenous proteases, thus improving resin–dentin bond durability. Application of crosslinking agents on demineralized dentin prevents the unwinding of triple-helical collagen, which consequently hinders MMPs from cleaving the collagen molecule polypeptides, thus increasing collagen degradation resistance and inactivating MMPs [7, 10–16].

It was reported that *Salvadora persica* extract includes many ingredients, among which are vitamin C and tannins [23, 27–29, 31, 32, 46–49]. Vitamin C has the ability to crosslink collagen I and III and act as an antioxidant and inhibitor of MMPs [50–52]. Antioxidants protect the structural integrity of the hybrid layer by counteracting the harmful effects of free radicals [53]. Furthermore, natural tannins are capable of crosslinking collagen fibrils, and Ghahri et al. [33] proved that chemical bonds are formed between amino acids and tannin constituents. However, to our knowledge, there is still no study that evaluated the effect of *Salvadora persica* on the durability of resin–dentin bonded interfaces.

Application of *Salvadora persica* for 60 s did not cause a significant change in the 24 h microtensile bond strength (*p* = 0.941) and nanoleakage values (*p* = 0.869) when compared to the control group. Thus, we should accept the first null hypothesis, which states that the use of *Salvadora persica* extract has no effect on immediate bond strength and nanoleakage.

Moreover, there was no significant difference in bond strength (*p* = 0.695) and nanoleakage (*p* = 0.437) in the 6-month specimens treated with *Salvadora persica* when compared to their 24 h controls. This result demonstrates improved stability of the bonded interfaces over the 6 m storage period, which comes in agreement with Khunkar et al. [28], who stated that *Salvadora persica* extract preserved the dentinal collagen from the collagenase enzyme. Thus, the second null hypothesis of the current study, which states that “the use of *Salvadora persica* extract has no preserving effect on the bonded interface over time”, should be rejected.

However, many variables can have a significant effect on bond strength, such as conditioning time [54], curing light [55], or type of adhesive system, whether self-etch or etch-and-rinse adhesive [2]. Therefore, future studies are needed, taking into careful account these variables in combination with the use of *Salvadora persica*. Moreover, the method of preparation of *Salvadora persica* extract [28, 29], its concentration as well as the time of application of the extract, may also play a significant role.

The results of the present study demonstrated a significant increase (*p* < 0.001) in the stiffness of demineralized dentin sticks after application of *Salvadora persica* for 1 min, from 4.93 ± 1.10 MPa to 9.32 ± 1.26 MPa (Fig. 4). This can be attributed to the possible crosslinking effect of *Salvadora persica*, which may increase the stiffness of the collagen network and consequently prevent the unwinding of the triple-helical collagen [56, 57]. Therefore, the third null hypotheses should be rejected which states that *Salvadora persica* has no reinforcing effect on dentin. Moreover, the 0.5 mm dentin sticks used in the stiffness test are much thicker than the acid-etched dentinal layers present clinically, which are nearly 10 μm thick [43]. Thus, it can be expected that 20% *Salvadora persica* extract can be applied for less time in the oral cavity. However, further research is required to validate this speculation.

Moreover, in the current study, *Salvadora persica* was able to reduce the amount of hydroxyproline release significantly (*p* < 0.001) when compared to the specimens that were placed in water (control). This may be due to increased collagen resistance to degradation and consequently inactivating dentinal MMPs, thus decreasing hydroxyproline release [13]. Consequently, we have to reject the fourth null hypothesis which states that *Salvadora persica* has no preserving effect on dentinal collagen by MMPs inactivation.

The current in vitro study had some limitations due to the difficulty of fully simulating the oral environment, including the absence of pulpal pressure, dentinal fluid, saliva, masticatory forces, pH, and temperature changes. Also, the design of specimens is not similar to that of the dental restorations that exist normally in the oral cavity.

## Conclusions

Within the limitations of the current study, it can be concluded that application of 20% *Salvadora persica* to acid-etched dentin for 1 min has maintained bond strength and nanoleakage over time without deterioration, as well as increasing stiffness and reducing collagen degradation by MMPs. Salvadora persica was efficient in preserving and enhancing the stability of resin–dentin bonded interfaces for 6 months. Further long-term trials using different variations of *Salvadora persica* and different application times are essential, aiming to improve the durability of esthetic restorations as well as bringing more outstanding applications of this unique biomaterial.

## Data Availability

All data are present in the manuscript, and raw data can be requested from the corresponding author forgenuine reasons.
